# Mapping of the Insomnia Severity Index and other sleep measures to EuroQol EQ-5D health state utilities

**DOI:** 10.1186/1477-7525-9-119

**Published:** 2011-12-30

**Authors:** Ning Yan Gu, Marc F Botteman, Xiang Ji, Christopher F Bell, John A Carter, Ben van Hout

**Affiliations:** 1 Pharmerit North America, LLC,4350 East West Highway, Suite 430, Bethesda, MD 20814, USA; 2 GlaxoSmithKline, Global Health Outcomes, Five Moore Drive, RTP, NC 27709, USA; 3 Pharmerit Ltd., Tower House, Suite 8, Fishergate - York, YO10 4UA, UK; 4 Department of Health Economics, HEDS, ScHARR, The University of Sheffield, Regent Court, 30 Regent Street, Sheffield S1 4DA, UK

**Keywords:** Insomnia, Mapping, Insomnia Severity Index, EQ-5D

## Abstract

**Background:**

This study sought to map the Insomnia Severity Index (ISI) and symptom variables onto the EQ-5D.

**Methods:**

A cross-sectional survey was conducted among adult US residents with self-reported sleep problems. Respondents provided demographic, comorbidity, and sleep-related information and had completed the ISI and the EQ-5D profile. Respondents were classified into ISI categories indicating no, threshold, moderate, or severe insomnia. Generalized linear models (GLM) were used to map the ISI's 7 items (Model I), summary scores (Model II), clinical categories (Model III), and insomnia symptoms (Model IV), onto the EQ-5D. We used 50% of the sample for estimation and 50% for prediction. Prediction accuracy was assessed by mean squared errors (MSEs) and mean absolute errors (MAEs).

**Results:**

Mean (standard deviation) sleep duration for respondents (N = 2,842) was 7.8 (1.9) hours, and mean ISI score was 14.1 (4.8). Mean predicted EQ-5D utility was 0.765 (0.08) from Models I-III, which overlapped with observed utilities 0.765 (0.18). Predicted utility using insomnia symptoms was higher (0.771(0.07)). Based on Model I, predicted utilities increased linearly with improving ISI (0.493 if ISI = 28 vs. 1.00 if ISI = 0, p < 0.01). From Model II, each unit decrease in ISI summary score was associated with a 0.022 (p < 0.001) increase in utility. Predicted utilities were 0.868, 0.809, 0.722, and 0.579, respectively, for the 4 clinical categories, suggesting that lower utility was related to greater insomnia severity. The symptom model (Model IV) indicated a concave sleep-duration function of the EQ-5D; thus, utilities diminished after an optimal amount of sleep. The MSEs/MAEs were substantially lower when predicting EQ-5D > 0.40, and results were comparable in all models.

**Conclusions:**

Findings suggest that mapping relationships between the EQ-5D and insomnia measures could be established. These relationships may be used to estimate insomnia-related treatment effects on health state utilities.

## Introduction

Insomnia is a disorder broadly defined by difficulty with sleeping. It may be characterized by 1) primary insomnia, without underlying medical cause; 2) secondary insomnia, with presence of an underlying medical cause; 3) acute insomnia, symptoms with a short duration or; 4) chronic insomnia, symptoms with a long duration [1,2]. Patients with insomnia commonly complain of difficulties initiating/maintaining sleep, early awakening, and non-restorative or poor quality sleep [3].

The prevalence of insomnia in the adult population ranges from 10% to 30% [1,4-6]. Insomnia is associated with substantial burden to patient and society. Persistent or prolonged sleeping problems have been associated with worsened health outcomes including reduced productivity or physical/social functioning, increased risk of occupational accidents or major depression/anxiety disorders, poorer health-related quality-of-life (HRQoL) and, increased health care costs [7-13]. Meanwhile, sleep-related conditions have often been under-diagnosed and under-treated [14].

A number of insomnia-related generic and disease-specific instruments have been used to identify and describe the condition. These instruments include, but are not limited to, the 36-Item Short-Form Health Survey [15], the Leeds Sleep Evaluation Questionnaire [16], the Medical Outcomes Study Sleep Scale 12 [17], the Epworth Sleepiness Scale [18,19], the Functional Outcomes of Sleep Questionnaire [20], the Pittsburgh Sleep Quality Index [21], and the Insomnia Severity Index (ISI) [22]. In addition to these instruments, insomnia symptom variables such as total sleep duration, sleep latency, number of nighttime awakenings, and the affect of prior night's sleep on next-day-sleepiness are predominant indicators of insomnia severity and are routinely collected in clinical studies of insomnia [7].

Among the various instruments used for describing insomnia, the ISI is one of the most commonly used disease-specific measures for self-perceived insomnia severity [23]. The ISI has 7 items describing insomnia-related health impairments concerning 1) difficulty falling asleep; 2) difficulty staying asleep; 3) waking up too early; 4) satisfaction with one's current sleep pattern; 5) self-perceived noticeability of current sleep problems to others with regard to patient's quality-of-life; 6) psychological burdens, and; 7) interference of sleep problems with one's daily functioning. Each item is rated on a 5-point Likert scale with scores ranging from 0 to 4, indicting "none", "mild", "moderate", "severe" and "very severe" sleep problems, respectively. The total ISI score is calculated by summing the scores from the 7 items, and range from a minimum of 0 to a maximum of 28, with higher scores reflecting more severe sleep problems. In clinical assessments, the ISI total summary score falls into 1 of 4 ISI categories; with scores 0-7, 8-14, 15-21, and 22-28 indicating no clinically significant insomnia, sub-threshold insomnia, moderate insomnia and, clinically severe insomnia, respectively. The psychometric properties of the ISI have been evaluated in earlier studies and have been reported to have sound measurement quality for measuring perceived insomnia severity and the impact of insomnia in different populations [22,24,25].

To quantify the impact of insomnia severity in economic studies such as cost utility analyses (CUA), preference-based measures are required to capture patient preferences for a particular health state [26]. Preference-based measures can be used to generate health state utilities based on a continuous scale whereby a utility of 1.00 represents "full" health and a utility score of 0.00 corresponds to "death". Such anchored scores are necessary to calculate quality-adjusted life-years (QALYs), a measure of life adjusted for the quality of that life, so that cross comparisons of different health care outcomes are permitted in health economic evaluations [26-28]. Following guidance issued by the National Institute for Health and Clinical Excellence (NICE, 2004) [29] in the United Kingdom, preference-based measures such as the EuroQol EQ-5D [30-32] or the Health Utility Indices [33] have become common means of generating health state utilities.

In particular, the EQ-5D is cognitively simple and takes only a few minutes to complete without imposing excessive response burden. It consists of five items describing health in terms of mobility, self care, usual activities, pain/discomfort, and anxiety/depression. Each item has 3 levels whereby higher levels indicate greater health deficits (1 = no problem, 2 = some problem and 3 = extreme problem). Hence, the EQ-5D descriptive system defines a total of 243 (3^5^) health states. Utility values can be computed from EQ-5D item responses using scoring algorithms [31,34]. Earlier studies have used the EQ-5D in insomnia-related studies for different populations, but mostly for secondary insomnia involving comorbid medical conditions such as depression or cancer [35,36].

CUAs have recently been conducted in the field of insomnia research [7,37-41]. Nonetheless, the evidence regarding the relationship between objective and subjective sleep measures and quantifiable insomnia-related health economic outcomes remains limited. In cases where direct evidence elicited by preference-based measures is not available, establishing a mapping relationship between descriptive clinical measures on insomnia and quantitative effects of insomnia on HRQoL can be useful.

The purpose of the present study was therefore to establish such a mapping relationship between insomnia-related measures and the EQ-5D. We aimed to estimate the associations between the EQ-5D health state utilities and insomnia severity measures by mapping the ISI and/or predominant indicators of insomnia onto the EQ-5D.

## Methods

### Survey

The analysis was based on a cross-sectional internet survey of approximately 3,000 US residents with signs and symptoms suggestive of chronic insomnia. This was an observational study designed to explore the relationship between subject-reported sleep measurements and outcomes (i.e. quality of life, functionality, and impact of sleep) in the US community. The survey was fielded by Harris Interactive which maintains a proprietary web-enabled panel of research subjects in the US who have agreed to participate in ongoing survey research.

Prior to the screening of any potential subjects, a central Institutional Review Board (IRB) approved the protocol (GHO-2008-008, 1/5/09), informed consent form, survey instruments, and all other subject information and/or recruitment materials. To recruit participants, e-mail invitations were sent to approximately 90,000 panel members representative of the general public. It was estimated that approximately 20% of the panel members in the specified subset would respond to the e-mail invitation. Of those, a 60% qualification rate was assumed among those insomnia-diagnosed patients. Overall, approximately 3,000 subjects were expected to enroll and complete the study. The study duration was estimated to be roughly 8 weeks, which included time for subject recruitment and completion of the questionnaire.

### Data

Subjects completed a questionnaire that collected information on demographics, comorbidities, and previous-night sleep symptoms. Subjects also provided responses to the ISI and the EQ-5D. Subjects with complete responses on the EQ-5D and the ISI questionnaire were included in the study if they a) were at least 18 years of age; b) provided informed consent to participate in the survey; c) were at least moderately bothered by their sleep problems; d) had reported problems with (i) falling asleep at the beginning of the night (ii) staying asleep throughout the night or (iii) not feeling refreshed upon waking following what was expected to be an adequate night's sleep for at least 3 times per week, or (iv) at least 2 of the problems listed above at least once per week.

Subjects were excluded from the study if they 1) were employed in full-time or part-time jobs that involved night shifts or day-night rotating shifts; 2) had children under 1 year old; or 3) reported a physician-diagnosis of competing symptoms of sleep such as obstructive sleep apnea, narcolepsy, periodic limb movement disorder, or restless leg syndrome.

### Models and Variables

A series of generalized linear models (GLM) was used for the present analysis. Based on the distribution of the variables, we indentified a gamma family distribution and a log link using the Modified Park tests for model specifications [42,43]. The dependent variable was the EQ-5D utilities computed based on the responses to the 5 items using a US algorithm [34]. While the gamma family was selected to account for the skewed dependent variable distribution, to respect its distribution for real values on a positive space (from 0 to ∞) [43], the modeled dependent variable was constructed as the disutility values of the EQ-5D (= 1-utility) computed using the following equation:

(1) Utility=1-Disutility=1- exp(α+ ∑Xiβ)

Four GLM functional forms were used (Table 1). For Models I-III the predictors for the EQ-5D disutility values were the 7 ISI items, a continuous (0-28) ISI summary score, and the 4 ISI clinical categories, respectively. For Model IV, we used sleep symptom variables identified from the existing literature on insomnia [7,44-46], namely, previous night's sleep duration, sleep quality, sleep latency, next-day-sleepiness as an effect of prior night's sleep, and the number of wakeup times during the night. Predictors in Model IV were supplemented with patient characteristics such as age, gender, and the presence/absence of comorbidities.

**Table 1:** Model overview

Dependent Variable (For Models I-IV)	Independent Variables and Models
	**Model I**: ISI 7 items $Utility_{\;\mathrm{mod}\;elI}=1-\exp\left(\alpha +\overset{7}{\underset{j=0}{\sum }}X_{i}\beta_{ij}\right)$*Where $\overset{0}{\underset{j=0}{\sum }}X_{i}\beta_{i0}\equiv 0$and, i = 1,...,7 representing 7 ISI items, j = 0,...,4, representing 5 levels of the Likert scale*.
	
	**Model II**: ISI summary scores (0-28) $Utility_{ModelII}=1-\exp\left(\alpha +X\beta \right)$*Where X is the ISI summary scores range from 0(min) to 28 (max) and treated as a continuous variable in the model*
	
EQ-5D disutility values Disutility= exp(α+ ∑Xβ) Utility = 1-Disutility	**Model III**: ISI 4 clinical categories $Utility_{ModelIII}=1-\exp\left(\alpha +\overset{4}{\underset{j=1}{\sum }}X\beta_{ij}\right)$*Where $\overset{1}{\underset{j=1}{\sum }}X_{i}\beta_{i1}\equiv 0$and, j = 1,...,4 representing 4 clinical classifications based on insomnia summary scores*.
	
	**Model IV**: Insomnia symptoms and demographics $Utility_{ModelIV}=1-\exp\left(\alpha +\overset{8}{\underset{i=1}{\sum }}X_{i}\beta_{i}+X_{duration^{2}}^{2}\beta_{duration^{2}}\right)$*Where i = 1,...,8 representing 5 symptoms, 2 demographic variables and 1 variable indicating the presence/absence of comorbidity. The 5 symptom variables were: sleep duration, quality, latency, next day sleepiness, and number of wakeup times during the night. The 2 demographic variables were age and gender. Within the model, a sleep duration squared term was an additional predictor adjusting the concave function of sleep duration on health state utilities*.

The comorbidity predictor was constructed as a binary variable representing the presence (= 1) or absence (= 0) of any of the 17 chronic non-insomnia-competing conditions reported by the respondents based on prior physician diagnoses. The chronic conditions included: anxiety disorder, arthritis, bipolar disorder, cancer, cardiovascular condition, chronic fatigue syndrome, chronic pain, depression, diabetes, drug/alcohol abuse, fibromyalgia, HIV/AIDS, insomnia, irritable bowel syndrome, neuropathic pain, respiratory condition, and schizophrenia.

The decision to use a single binary(yes/no) comorbidity presence indicator rather than one variable per condition or counting the sum of the total number of conditions was made, primarily, to impose a minimal burden on future data collection. Specifically, it should be emphasized that the objective of this study was not to predict utility levels using a large number of clinical and demographic variables. Rather, we sought to construct a simple--if not generic--tool that would allow researchers to predict utility in a community-based population of individuals exhibiting insomnia symptoms using as few variables as possible. Ultimately, we hope that the algorithm generated in this process can be used by researchers who either could not collect utilities in previous research or, for other reasons, will not be able to do so in the future. Hence, the focus of this analysis was on external rather than internal validity. The approach selected herein with regard to comorbidity was consistent with the broader sleep-research literature which emphasizes insomnia without comorbidities (i.e., primary insomnia) from insomnia with comorbidities (i.e., secondary insomnia).

For the sleep duration variable used in the Model IV, based on preliminary analysis of the predictors, observed EQ-5D health state utility was found to be optimal when the amount of sleep was approximately 7-9 hours (mean [standard deviation, SD] sleep duration = 7.8 [1.9] hours). EQ-5D utility decreased when one slept more/less than that optimal amount or extreme hours, which gave a concave sleep duration function of EQ-5D utility/disutility. Thus, a squared term of the sleep duration variable was included in the model.

Moreover, the sleep quality-rating variable ranged from 0, indicating poor sleep quality to 10, indicating excellent sleep quality. The next-day-sleepiness item also used a rating ranging from 0 suggesting not feeling sleepy due to prior night's sleep pattern to 10 for feeling extremely sleepy. Sleep latency was captured using total minutes of delay to sleep and the total number of wake up times during the night ranged from minimum of 1 time to a maximum of 5 times. We treated these predictors as continuous for simplicity.

### Analyses

We used 50% of the sample to generate the mapping function (i.e., estimation sample) and, the other 50% to validate the model performance (i.e., validation sample). Samples were randomly split for each process. Predictions in the validation process were made based on parameters estimated from estimation sample. The validation process was repeated 30 times to ensure we obtained a sufficient number of predictions to account for variability. Average values from the 30 validations were calculated for each of the 4 models.

To determine the predictive precision of the models, we computed model mean square error (MSE) and mean absolute error (MAE). The MSE was given by: $MSE=\frac{\sum {\left(Y-Ŷ\right)}^{2}}{N-K}$where Y = observed EQ-5D values,  = predicted EQ-5D values, N = total or subgroup sample sizes and, K = degrees of freedom. Hence, the MSEs were computed by adjusting the number of independent variables included in the model. Thus, a perfect prediction would be indicated by a zero MSE and, smaller MSEs indicated lower prediction errors. Since MSEs penalized larger errors by using the squared term of errors, we also computed the MAE. The MAE was given by: $MAE=\frac{\sum \left|Y-Ŷ\right|}{N}$, again, Y = observed EQ-5D values,  = predicted EQ-5D values N = total or subgroup sample sizes, The MAEs provided error statistics that did not give greater weight to larger errors.

The overall model performance was also assessed by examining the distributional qualities of the predicted EQ-5D scores compared with the observed ones with regard to mean, median, min/max, and range. SAS 9.2 was used for data preparation, STATA11 was used for regression and statistical tests, and Microsoft Excel was used for prediction error computations.

## Results

Of the 3,034 survey participants, 2,842 (93.67%) met all inclusion/exclusion criteria and were included in the present analyses. Table 2 reports respondents' socio-demographic characteristics. The mean (± SD) age of the total sample was 42.9 (± 15.7) years. Nearly two thirds of respondents were female and 83.7% were Caucasian. The mean (± SD) observed EQ-5D utility score was 0.765 (± 0.20) and was lowest for individuals aged 50 to 59 (0.749 [± 0.20]). On average, respondents reported 7.8 (± 1.9) hours of sleep during the previous night. The mean (± SD) ISI summary score was 14.1 (± 4.8). According to the ISI clinical classification, 6.19%, 52.08%, 33.57%, and 8.16% of respondents were categorized as having no clinically significant insomnia, sub-threshold insomnia, moderately severe insomnia, and severe insomnia, respectively. Individuals aged 40 to 59 reported the least mean (± SD) amount of sleep duration (7.5 [± 1.8]) and higher scores on ISI items, suggesting greater sleep problems. In addition, compared with male respondents, females reported longer mean sleep durations (7.9 vs. 7.5 hours) and higher mean EQ-5D scores (0.766 vs. 0.762) despite higher ISI summary scores (14.3 vs. 13.7). In this particular sample 62.4% of respondents reported ≥ 1 comorbid medical conditions and, for those who reported ≥ 1 comorbid conditions, mean EQ-5D scores were lower compared with those who reported no comorbid conditions (0.723 vs. 0.835). ISI summary scores decreased (i.e., insomnia improved) as sleep quality improved, and increased (i.e., insomnia worsened) with rating increases of next-day-sleepiness, sleep latency, and number of wake-up times. Therefore, a longer time to fall asleep and a greater number of wake-up times during a night were associated with higher ISI scores and lower the EQ-5D utility values.

**Table 2:** Sample Characteristics

	Number	%	Mean Age (SD) (Years)	Mean (SD) EQ-5D	Mean Duration (SD) (Hours)	Mean ISI (SD)
Whole sample	2,842	100	42.9 (15.7)	0.765 (0.2)	7.8 (1.9)	14.1 (4.8)

Age group						

18-29	743	26.1	24.2 (3.8)	0.784 (0.2)	8.0 (1.9)	13.4 (4.6)

30-39	569	20.0	34.7 (2.9)	0.754 (0.2)	7.7 (1.9)	15.0 (5.0)

40-49	624	22.0	45.0 (2.8)	0.754 (0.2)	7.5 (1.8)	15.0 (4.7)

50-59	405	14.3	54.9 (2.8)	0.749 (0.2)	7.5 (1.8)	14.2 (4.7)

60-69	351	12.4	64.2 (2.9)	0.770 (0.2)	7.9 (1.9)	12.9 (4.5)

≥ 70	150	5.30	74.8 (4.6)	0.786 (0.1)	8.1 (1.6)	12.3 (4.7)

Gender						

Female	1,834	64.50	42.0 (15.9)	0.766 (0.2)	7.9 (1.9)	14.3 (4.8)

Male	1,008	35.50	44.5 (15.2)	0.762 (0.2)	7.5 (1.8)	13.7 (4.7)

Race						

White	2,318	83.7	44.0 (15.7)	0.768 (0.2)	7.8 (1.8)	13.9 (4.7)

Black	292	10.5	38.1 (14.7)	0.755 (0.2)	7.4 (2.1)	14.7 (5.1)

Asian	44	1.60	26.7 (10.4)	0.805 (0.1)	7.4 (2.1)	13.7 (3.7)

Other	116	4.20	38.3 (13.7)	0.719 (0.2)	7.5 (2.1)	15.7 (5.3)

Comorbidity						

No	1068	37.60	38.2 (14.4)	0.835 (0.1)	7.7 (1.8)	12.7 (4.3)

Yes	1774	62.40	45.7 (15.8)	0.723 (0.2)	7.8 (1.9)	14.9 (4.9)

Sleep quality						

0	146	5.14	43.6 (13.4)	0.614 (0.2)	6.6 (2.5)	20.5 (5.1)

1	94	3.31	43.7 (15.7)	0.697 (0.2)	7.2 (2.3)	18.3 (4.8)

2	250	8.81	43.9 (14.2)	0.712 (0.2)	7.4 (2.2)	17.1 (4.6)

3	457	16.10	44.0 (15.0)	0.755 (0.2)	7.7 (1.9)	15.5 (4.1)

4	457	16.10	41.6 (14.8)	0.763 (0.2)	7.9 (1.8)	13.9 (4.0)

5	559	19.70	43.3 (15.7)	0.795 (0.1)	7.8 (1.6)	12.9 (3.9)

6	411	14.48	40.9 (16.8)	0.800 (0.1)	8.0 (1.6)	12.2 (3.7)

7	291	10.25	42.7 (17.0)	0.804 (0.2)	8.0 (1.7)	11.6 (4.1)

8	108	3.81	43.8 (17.8)	0.781 (0.2)	8.4 (1.8)	10.9 (4.0)

9	35	1.23	40.5 (18.3)	0.858 (0.2)	8.5 (1.2)	11.7 (5.2)

10	30	1.06	48.3 (17.6)	0.727 (0.3)	8.0 (2.2)	10.5 (5.7)

Next day sleepiness						

0	162	5.71	54.0 (15.8)	0.764 (0.2)	7.8 (2.0)	12.4 (5.4)

1	100	3.52	49.3 (16.6)	0.805 (0.2)	8.0 (2.0)	12.2 (4.5)

2	157	5.53	45.0 (16.9)	0.792 (0.2)	7.9 (1.8)	12.5 (4.0)

3	213	7.51	46.0 (16.0)	0.788 (0.2)	7.8 (1.7)	12.4 (4.1)

4	236	8.32	44.6 (16.1)	0.776 (0.2)	7.8 (1.8)	13.0 (4.1)

5	373	13.15	44.4 (15.6)	0.781 (0.2)	7.9 (1.8)	13.5 (4.3)

6	436	15.37	40.9 (15.4)	0.783 (0.2)	7.8 (1.9)	13.5 (4.2)

7	520	18.33	40.6 (14.2)	0.758 (0.2)	7.8 (1.8)	14.7 (4.6)

8	364	12.83	38.4 (14.3)	0.747 (0.2)	7.7 (1.9)	15.6 (4.8)

9	135	4.76	40.5 (13.7)	0.683 (0.2)	7.5 (2.1)	16.2 (5.3)

10	141	4.97	39.2 (14.0)	0.710 (0.2)	7.2 (2.2)	18.1 (5.5)

Latency (minutes)						

< 15	443	17.39	44.2 (15.2)	0.808 (0.1)	7.7 (1.7)	11.9 (4.3)

15-30	468	18.37	44.7 (16.5)	0.799 (0.2)	7.8 (1.7)	12.3 (4.1)

30-45	449	17.62	42.9 (16.0)	0.791 (0.1)	7.8 (1.7)	13.5 (4.3)

45-60	205	8.05	41.8 (15.0)	0.773 (0.2)	7.6 (1.7)	13.9 (4.3)

60-90	404	15.86	42.3 (15.1)	0.752 (0.2)	7.8 (1.8)	14.6 (4.4)

> = 90	579	22.72	40.5 (15.9)	0.729 (0.2)	8.0 (2.1)	15.9 (4.7)

Wake up times						

1	375	18.20	43.6 (15.4)	0.802 (0.2)	7.5 (1.7)	12.8 (4.3)

2	732	35.6	44.5 (16.1)	0.780 (0.2)	7.7 (1.7)	13.6 (4.5)

3	588	28.6	44.1 (15.6)	0.759 (0.2)	8.0 (1.8)	14.3 (4.4)

4	184	8.90	44.9 (15.2)	0.743 (0.2)	8.3 (1.8)	15.6 (4.7)

5	179	8.70	42.6 (13.0)	0.715 (0.2)	8.4 (1.8)	16.2 (4.6)

Table 3 exhibits the model regression estimates using the different set of predictors on EQ-5D disutility. For Model I, except for ISI item 4 (i.e., degree of satisfaction with sleep pattern) and item 7 (extent of interference with daily functioning due to sleep pattern), ISI items showed significant associations with EQ-5D scores (p < 0.05). Higher levels of ISI items were associated with greater health deficit (greater disutility values). ISI summary scores in Model II and clinical classification scores in Model III also showed significant associations with EQ-5D disutility values (p < 0.001). In Model IV, except for age and gender, all other predictors were able to provide substantial explanations of EQ-5D scores (p < 0.05). The signs of model coefficients were as expected.

**Table 3:** Model Estimations

	Model I	Model II	Model III	Model IV
Intercept	-2.45614 (0.27103) ****	-2.34909 (0.0493) ****	-2.0259 (0.0733) ****	-0.99338 (0.17896) ****

ISI_1				

1	0.04681 (0.05083)	--	--	--

2	0.14743 (0.04637) ***	--	--	--

3	0.23814 (0.05315) ****	--	--	--

4	0.20228 (0.08007) **	--	--	--

ISI_2				

1	-0.00487 (0.06257)	--	--	--

2	0.10069 (0.05168) *	--	--	--

3	0.1438 (0.0581) **	--	--	--

4	0.22504 (0.08002) ***	--	--	--

ISI_3				

1	-0.04075 (0.04682)	--	--	--

2	0.00373 (0.04365)	--	--	--

3	0.13501 (0.05178) ***	--	--	--

4	0.15058 (0.06278) **	--	--	--

ISI_4				

1	0.30681 (0.28409)	--	--	--

2	0.23039 (0.26934)	--	--	--

3	0.14912 (0.26789)	--	--	--

4	0.13552 (0.26478)	--	--	--

ISI_5				

1	0.18721 (0.12667)	--	--	--

2	0.3242 (0.12139) ***	--	--	--

3	0.41963 (0.12265) ***	--	--	--

4	0.58239 (0.12811) ****	--	--	--

ISI_6				

1	0.13325 (0.0471) ***	--	--	--

2	0.16124 (0.04784) ***	--	--	--

3	0.24329 (0.05675) ****	--	--	--

4	0.38139 (0.06977) ****	--	--	--

ISI_7				

1	0.04661 (0.07847)	--	--	--

2	0.0766 (0.07898)	--	--	--

3	0.11301 (0.0838)	--	--	--

4	0.16077 (0.09968)	--	--	--

ISI summary	--	0.06077 (0.00307) ****	--	--

ISI class				

2	--	--	0.36953 (0.07558) ****	--

3	--	--	0.74122 (0.07544) ****	--

4	--	--	1.15809 (0.08459) ****	--

Age	--	--	--	-0.00079 (0.00103)

Female	--	--	--	-0.03204 (0.02736)

Duration	--	--	--	-0.22818 (0.04158) ****

Duration^2^	--	--	--	0.0133 (0.00265) ****

Comorbidity	--	--	--	0.42901 (0.02861) ****

Sleep quality	--	--	--	-0.0314 (0.00953) ***

Latency	--	--	--	0.00112 (0.00026) ****

Sleepiness	--	--	--	0.02498 (0.00754) ***

Wake times	--	--	--	0.05213 (0.01348) ****

Log-likelihood	1378.765	1405.107	1392.894	1018.948

For model specifications please refer to Table 1

ISI class_1 = No clinically significant insomnia; ISI class_2 = Subthreshold insomnia; ISI class_3 = Moderate insomnia; ISI class_4 = Se were insomnia

* p < 0.10; ** p < 0.05; *** p < 0.01; **** p < 0.001

Table 4 compares the mean, median, min/max, and range of observed and predicted EQ-5D values for both estimation and validation sample estimates. EQ-5D utility values were computed using equation (1). The observed EQ-5D mean (± SD) utility value was 0.765 (± 0.18), with a median value of 0.800 and a range of [-0.040-1.000]. Mean predicted scores were identical with those observed for three ISI models (Models I-III). The predicted mean was slightly higher for Model IV, when insomnia symptoms were used for prediction (0.771 (± 0.07)), compared with Models I-III using ISI (0.765 (± 0.08)). Similar results were found in validation sample estimates.

**Table 4:** Comparison of observed and predicted EQ-5D utility values

		Mean (SD)	Median	[Min-Max]	Range
	
	Observed	0.765 (0.18)	0.800	[-0.040-1.000]	1.040
Estimation Sample	Model I	0.765 (0.08)	0.783	[0.407-0.914]	0.507
	
	Model II	0.765 (0.07)	0.776	[0.477-0.905]	0.428
	
	Model III	0.765 (0.07)	0.809	[0.580-0.868]	0.288
	
	Model IV	0.771 (0.07)	0.772	[0.324-0.895]	0.571

Validation Sample	Model I	0.766 (0.08)	0.786	[0.367-0.891]	0.524
	
	Model II	0.765 (0.07)	0.776	[0.488-0.896]	0.408
	
	Model III	0.764 (0.07)	0.811	[0.571-0.858]	0.287
	
	Model IV	0.774 (0.07)	0.777	[0.321-0.906]	0.585

For model specifications please refer to Table 1

Table 5 shows the model prediction errors. The MSEs and MAEs were lower for EQ-5D utilities greater than 0.40. This suggested that predictions were more robust for higher health state utilities (i.e., > 0.4), which were reported by 94.02% of the respondents in the sample. At the same time, relatively poorer predictions at tail end of utilities have been reported as a common problem associated with mapping [47]. Owing to the very small sample size (0.63%) of respondents who had observed utilities < 0.2, MSEs for EQ-5D utilities less than 0.2 could not be computed in Models I and IV. Similar results were found using the validation samples.

**Table 5:** Assessing model predictions

		N	Model I		Model II		Model III		Model IV	
			
			MSE	MAE	MSE	MAE	MSE	MAE	MSE	MAE
Estimation Sample	Full index	1421 (100%)	0.024	0.109	0.025	0.110	0.026	0.112	0.024	0.110
	
	< 0.2	10 (0.68%)	N/A	0.545	0.375	0.566	0.424	0.567	N/A	0.526
	
	0.2-0.4	53 (3.76%)	0.184	0.350	0.146	0.366	0.155	0.377	0.192	0.392
	
	0.4-0.6	164 (11.57%)	0.070	0.232	0.066	0.235	0.068	0.241	0.072	0.250
	
	0.6-0.8	329 (23.15%)	0.008	0.066	0.007	0.063	0.007	0.064	0.005	0.057
	
	0.8-1.0	865 (60.85%)	0.013	0.083	0.013	0.084	0.014	0.085	0.013	0.087

Validation Sample	Full index	1421 (100%)	0.025	0.110	0.024	0.110	0.025	0.111	0.023	0.108
	
	< 0.2	10 (0.73%)	N/A	0.538	0.351	0.555	0.389	0.547	N/A	0.502
	
	0.2-0.4	53 (3.70%)	0.209	0.373	0.157	0.381	0.165	0.388	0.207	0.407
	
	0.4-0.6	163 (11.44%)	0.071	0.233	0.064	0.234	0.067	0.235	0.071	0.246
	
	0.6-0.8	326 (22.95%)	0.008	0.069	0.007	0.061	0.007	0.064	0.006	0.061
	
	0.8-1.0	869 (61.18%)	0.013	0.083	0.013	0.084	0.014	0.085	0.013	0.087

-MSE = Mean square error.

-N/A = Estimates were not available due to the small sample size

-MAE = Mean absolute error

- Note: For model specifications please refer to Table 1

-Sample size for both the estimation and validation sample was randomly drawn (50%) from the total qualified participants (N = 2,842).

**Figure 1:**
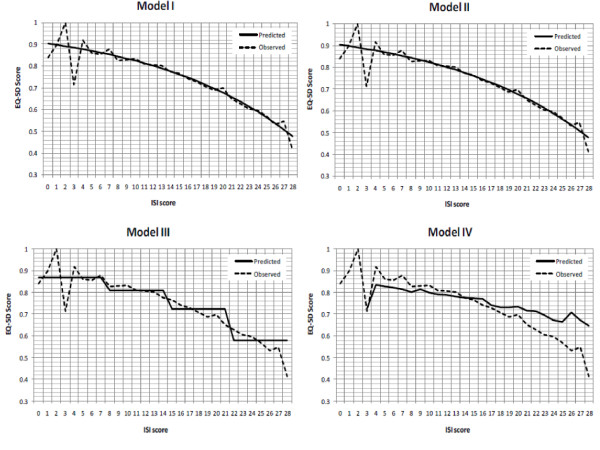
**Observed and predicted EQ-5D utility values along ISI scores**. Note: For model specifications please refer to Table 1.

Figure 1 depicts observed and predicted EQ-5D utility values for Models I-IV along the ISI scores (ranging from 0 to 28), which showed negative correlations between the two instruments. The predicted EQ-5D utility values followed more closely with observed ones for mid-range scores, especially for Models I-III. For Model IV, EQ-5D utility values were under-predicted for ISI scores less than 14 (no insomnia or threshold insomnia) and over-predicted for ISI scores greater than 14 (moderate or severe insomnia). Nevertheless, the association between greater insomnia symptom severity and lower health utilities was observed in all models.

**Figure 2:**
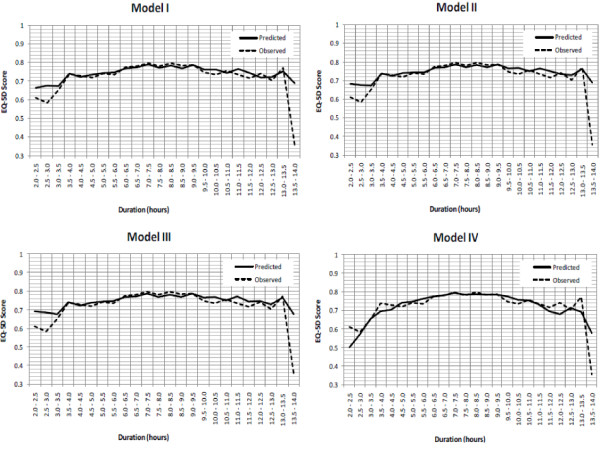
**Observed and predicted EQ-5D utility values along sleep duration (in half-hour intervals)**. Note: For model specifications please refer to Table 1.

Figure 2 shows the observed and predicted EQ-5D values along sleep duration using half-hour intervals. Again, predicted and observed EQ-5D utilities followed each other closely along sleep durations except for the higher and lower ends of sleep hours (i.e., extreme hours). The concave function of sleep duration of the EQ-5D utility was preserved, suggesting that health state utilities increase at a decreasing rate along sleep durations.

## Discussion

The extent of association between a disease-specific instrument (i.e., ISI) and a generic preference-based instrument (i.e., EQ-5D) is affected by the amount of correspondence between the two measures with regard to the underlying HRQoL [48]. Findings from this analysis suggest there was sufficient overlapping of underlying HRQoL between insomnia measures and EQ-5D health state utilities. While the ISI is commonly used in clinical trials and was specifically designed to describe and evaluate insomnia severity, it does not provide the preference-based measures necessary for health economic evaluations quantifying the impact of insomnia on patients' health state utilities. To the best of our knowledge, the present study is the first to map an insomnia-specific instrument and/or sleep variables to EQ-5D utility values. Hence, the cross-walk conducted herein establishes a preliminary mapping relationship and link between these measures.

Findings from this analysis were consistent with earlier studies wherein poorer sleep outcomes (e.g., sleep quality, next-day-sleepiness, and extreme sleep hours) were significantly associated with declining HRQoL [8,12,49]. Such associations were observed in the present analysis, especially for individuals with greater sleep difficulties (Figure 1) or extreme sleep durations (Figure 2).

With respect to sleep duration for example, a recent study by Faubel et al. (2009) reported that for older men and women, extreme sleep durations (≤ 5 hours or ≥ 10 hours) were indicators of worse HRQoL, compared with those who sleep 7-8 hours per night [8]. Studies of different populations have also reported this same effect: that short-duration sleep [50] or long-duration sleep [51] or both [52], were associated with worse HRQoL. This association was noted in our model predictions by taking other factors into consideration (Model IV).

Quantifying the effect of insomnia on HRQoL is essential for targeting effective treatments, and comparing treatment cohorts in clinical practice. The mapping relationship established between the ISI and the EQ-5D in this analysis provides a necessary cross-walk when a preference-based measure such as EQ-5D was not administered. For example, using a hypothetical scenario where a 36-year old female had ISI item scores of 0, 3, 1, 4, 3, 3, and 2, which would result in an ISI summary score of 16 and a ISI class of 3. Further, she also reported 8.58 hours of sleep, ≥ 1 comorbidity, 1 on overall sleep quality, 5 minutes of sleep onset latency during previous night, 8 on next day sleepiness, and 5 wake-up times. Using the algorithms provided (Table 1 equation (1)), her estimated EQ-5D utility values would be 0.772, 0.748, 0.723, and 0.690 using Models I-IV, respectively (see Table 6 for sample computations).

**Table 6:** Sample utility computation

Model	EQ-5D utility	Computation
I	0.772	≈1-exp(-2.45614+0+0.14380-0.04075+0.13552+0.41963+0.24329+0.0766)

II	0.748	≈1-exp(-2.34909+ 0.06077*16)

III	0.723	≈1-exp(-2.0259+ 0.74122)

IV	0.690	≈1-exp(-0.99338-0.00079*36-0.03204*1-0.22818*8.58+0.0133*8.58^2^+0.42901*1-0.0314*1+0.00112*5+0.02498*8+0.05213*5)

Sample scenario:

Assume a 36-year old female had ISI scores of 0, 3, 1, 4, 3, 3, and 2 on the 7 items, respectively, resulting in an ISI summary score of 16 and a ISI class of 3, in combination with a report of 8.58 hours of sleep, ≥ 1 comorbidity, 1 on overall sleep quality, 5 minutes of sleep onset latency during previous night, 8 on next day sleepiness, and 5 wake up times.

For this particular scenario, compared with Model I, the estimated utilities using Models II and III were decreased by approximately 3% and 6%, respectively (0.772 vs. 0.748 and 0.723). When Model IV was used, the estimated utility was lower by 10% (0.772 vs. 0.690). These discrepancies were associated with different model specifications as each model incorporated different assumptions. In Model I, the predictions based on 7 distinctive item scores were able to capture more precise information for describing the EQ-5D and different items categories were assumed to have their own weight on the EQ-5D scores. Conversely, in Model II a continuous summary score ranging from 0 to 28 was used. This assumed that each unit increment/decrement in total ISI score would give the same impact on the EQ-5D regardless of which item. The case is similar for Model III, where the clinical categories were constructed based on the summary scores. One way to conceptualize this is to understand that Models II and III penalized the estimated utilities by placing more weight on the items that had more impact on the EQ-5D. Model IV is a different model specification aimed to estimate the same outcome. It captured more disease-specific information in explanatory variables, and therefore the estimated EQ-5D utilities were penalized the most. Alternatively, these discrepancies could be lowered by using a sample scenario with all best levels of ISI items where no problems were reported on all items, i.e., score = 0 on all items. Using the same computation, the estimated utilities for Models I-III would be approximately 0.914, 0.905 and 0.868, respectively, resulting 1% and 5% differences in estimated utilities using Models II and III, respectively compared with Model I. Given these findings, Model I should be used when data for all ISI items are available.

Based on the findings, marginal changes on treatment effects on insomnia could also be estimated. If the same respondent had improved her sleep pattern somehow and decreased her ISI score to 14 (from 16), then by using Model II, her predicted EQ-5D utility score would be 0.776 (= 1-exp(-2.34909+ 0.06077* 14) instead of 0.748, corresponding to a difference of approximately 0.0289 unit of utility. On the other hand, holding other variables constant and assuming changes to ISI item 5 (e.g., if she thought her sleep problem was noticed "a little by others" (level 1)); then using the Model I algorithm, her health utility would be 0.761 (= 1-exp(-2.45614+0.04681+0.14380-0.04075+0.13552+0.41963+0.24329+0.0766)), instead of 0.772. Similar magnitudes of health disutility gains and losses could be estimated if changes had taken place on other ISI items. From a health policy standpoint, such quantification is useful for assessing the effect of treatment on health utility values. Thus, the findings provided herein make it possible to estimate EQ-5D utility values when direct evidence is absent from the primary research, but when ISI or insomnia-related symptoms data are available.

It is important to note the limitations of this study. First, while significant associations were noted between insomnia measures and EQ-5D utilities in the current study, the mapping technique implicitly assumes that the EQ-5D covers all important aspects of the latent health construct that the ISI is intended to measure. Hence, the strength of the mapping function is underpinned by the degree of overlap between the two measures. While all model estimates rendered very close approximations of the EQ-5D observed scores, the regression-based transformation presented herein is self-contained in that disease-specific scores are permitted to be transformed to a generic utility measure without referencing additional data and aimed for repeated uses on secondary data analysis from other clinical trials [48]. Therefore, the external validity of the study should be verified using other data sets.

Second, since different models control for different predictors, each entails a different magnitude of effects. At the group level, predicted mean utility values closely estimated observed values. At the individual level, algorithms presented in this study may include some discrepancies from one model to another, as was indicated in the above example. Therefore, our algorithms may more accurately predict individual EQ-5D values for a group level evaluation for CUA studies in making group level comparisons.

Third, regression-based mapping from one measure to another inevitably results in floor and ceiling effects for predicted values [48]. We noted higher MSEs in all models for lower EQ-5D utility values (EQ-5D < 0.4) Thus, our algorithms were less reliable for respondents with EQ-5D scores < 0.4.

Fourth, QALYs implicitly involve the concept of survival, but we estimated health utility values with a cross-sectional data set. Hence, the directional effects of insomnia on patients' utility over time, or, the responsiveness of the estimates could not be assessed in the present study. It is therefore unclear whether the mathematical links reported in this study would vary over time.

Fifth, the current study relied on respondents' self-reported health status and not on physician assessment or medical records. Hence, the aim was not to identify cause-and-effect relationships between sleep problems and other specific conditions or parameters. Interestingly, findings based on responses from such a convenient sample and not from a controlled clinical study were strikingly consistent with the study hypotheses, which provided vital validity of the analyses.

Sixth, treating the sleep quality variable in Model IV as continuous was a simplistic approach which assumed that each unit increase in sleep quality equally impacted the EQ-5D. Comparisons on the prediction results using the continuous and the categorical sleep quality showed that both approaches had equivalent predicted outcomes; although, the trade-off was that distinctive effects on each sleep quality categories on the EQ-5D could not be separated under the continuous approach. Since the purpose of this study was to obtain the best estimates of the EQ-5D based on pre-defined predictors, we reported our findings under the continuous approach in Model IV.

Finally, the binary indicator of comorbidity used herein disregarded the possibility that a respondent with more than one comorbidity would likely report a worse utility, all other things being equal, than a respondent with one comorbidity. Likewise, this approach did not take into account which comorbidity was present, thus ignoring the possibility that some condition may have had a stronger impact on utility than others. It was beyond the scope of the present analysis to capture a more detailed quantification of these effects. Rather, an approximation of the EQ-5D utility values based on included parameters could be implemented using Model IV. Based on our findings, such approximation or estimation could be carried out in patients with either no comorbidities (i.e., primary insomnia) or in patients with one or more comorbidities (i.e., secondary insomnia). More importantly, the model was implementable regardless of whether the comorbidity conditions were listed in the current survey.

## Conclusion

Estimating EQ-5D health state utility values using the algorithms presented herein permits comparisons of health outcomes in the absence of preference-based measures. Despite the aforementioned limitations, these algorithms give flexibility of computing EQ-5D health state utilities using different types of empirical insomnia data. Meanwhile the mapping relationship explored in this study serves as a "second-best" approach relative to direct elicitation of preference-based measures in clinical studies. Out-of-sample validation of these algorithms is encouraged to further establish the relationship between insomnia measures and the EQ-5D. This is especially true among patient groups with relatively lower observed health state utility values.

## Competing interests

This manuscript was funded by GlaxoSmithKline. CFB is an employee of GlaxoSmithKline. NYG, MFB, XJ, JAC, and BvH are employees of Pharmerit which was paid a consulting fee by GlaxoSmithKline related to the development of this manuscript.

## Authors' contributions

NYG, MFB, and BvH contributed to study design, analysis, interpretation, and manuscript writing. XJ and CFB contributed to study design, analysis, and interpretation. JAC contributed to analysis, interpretation, and manuscript writing. All authors have read and approved the final manuscript.
